# The Role of Applied Behavior Analysis to Improve Knowledge on Oral Hygiene Practices among Cooperative Autistic Children: A Cross-Sectional Study from Jazan, Saudi Arabia

**DOI:** 10.1155/2021/9491496

**Published:** 2021-07-20

**Authors:** Hytham N. Fageeh, Manawar A. Mansour, Hatim Y. Thubab, Mohammed B. Jarab, Ahmed Y. Juraybi, Hassan H. Zakri, Abdullah M. Bahri

**Affiliations:** ^1^Department of Preventive Dental Sciences, College of Dentistry, Jazan University, Jazan 45412, Saudi Arabia; ^2^Department of Prosthetic Dental Sciences, College of Dentistry, Jazan University, Jazan 45412, Saudi Arabia; ^3^College of Dentistry, Jazan University, Jazan 45412, Saudi Arabia

## Abstract

**Objective:**

To assess the effectiveness of Applied Behavior Analysis (ABA) to improve knowledge regarding oral hygiene practices among cooperative autistic children.

**Materials and Methods:**

A cross-sectional study was conducted among 15 children between the age group of 6–12 years and their parents who were randomly chosen from a special care autistic school in Jazan, Saudi Arabia. A mobile application was custom designed and programmed with videos on oral hygiene. A close-ended questionnaire comprising 14 questions for the cooperative autistic children and 21 questions for their parents was designed to assess their knowledge in relation to oral health and hygiene. After four weeks, a questionnaire-based knowledge assessment was conducted. The mean knowledge score was then calculated for children and their parents and compared using paired sample *t*-test.

**Results:**

Poor knowledge regarding oral hygiene practices was revealed among the study participants. The estimated mean score among the children was 4.73 before the intervention, which significantly increased to 9.0. The estimated mean score for the parents was 9.3 before intervention and 14.6 after four weeks' period (*P* < 0.0001).

**Conclusion:**

The application of ABA using avatars and delivered through videos can significantly improve knowledge regarding oral health hygiene among cooperative autistic children.

## 1. Introduction

Autism or autism spectrum disorder (ASD), the most common neurodevelopmental disorder, is delineated by three prominent features: language delay, hindered communication interaction, and monotonous patterns of behavior. Environmental exposures and genetic factors are known to be associated with autism [1]. According to the World Health Organization (WHO), the prevalence of autism spectrum disorder (ASD) is 1 per 160 children globally [2], 21.6 in the US, 18.75 in Europe, and 11.6 per 10,000 children in China [3, 4]. Reports indicate an increase in autism prevalence even in Arab countries with 1.4 in Oman, 29 in UAE, and 59 per 1000 children in Saudi Arabia [5–10].

General health, including oral health, is usually neglected in autistic children due to a multitude of factors. These children manifest increased sensitivity to sounds, light, odours, and colours, an attribute that challenges the attainment of good oral hygiene. The fear of the unknown and their sensory hypersensitivity hinder examination of oral cavity and treatment if required [11, 12]. Additionally, dental care for special needs patients is often neglected due to the paucity of dentists specialized in treating such individuals and due to difficulty managing these children's behavior [13]. Consequently, these children are at risk of developing dental and oral diseases due to poor dietary habits, damaging oral habits such as bruxism, and oral self-injures [14].

Several communication-aided approaches, such as applied behavioral analyses, visual pedagogies, pictorial or iconic images, and social stories, have been proposed to aid behavioral changes among people with ASD [15–18]. Special educators and therapists have opined that early intensive, continuous special educational programs and behavioral therapies modify autistic children's behavior and help them in achieving better self-care, social, and communication skills [17, 18].

Over the last decade, researchers have designed and evaluated numerous digital solutions (e.g., games, learning applications, tabletop applications, and robotic solutions) for assisting autistic children of different age groups [19–24]. Avatars have been used in a recent study to enable autistic children to understand human emotions and expressions [22, 24]. Despite an immense amount of autism research, the role of culture while designing rehabilitation applications has relatively been understudied. Similarly, most of the research on ASD is limited to children from western countries [24, 25].

The present study was conducted to assess the effectiveness of the ABA (Applied Behavior Analysis) delivered through Avatar technology to improve knowledge of oral hygiene practices among autistic children. The null hypothesis states that there would be no differences in the knowledge related to oral health hygiene practices among autistic children and their parents using ABA delivered through avatars.

## 2. Materials and Methods

### 2.1. Study Design and Population

A cross-sectional study was conducted among 15 cooperative autistic children between the age group of 6–12 years, randomly chosen from the special care autistic school (Aafiya Special Needs School), and their parents in Jazan, Saudi Arabia, to achieve the objectives of the study. The inclusion criteria were the willingness and approval of the autistic children and their parents to participate in the study. Any autistic child aged 6–12 who does not show a sign of or known to be/have any of the following: uncooperative autistic children or their parents, children under medications affecting gingival or periodontal health, and any previous history of dental visits were excluded from the study.

### 2.2. Informed Consent and Ethical Clearance

The purpose of the study was explained to the participants (the children and their parents), and written informed consent was obtained from the parents. The study was conducted according to the guidelines of the Helsinki declaration, and ethical clearance was obtained from the Standing Committee for Scientific Research Ethics at Jazan University (Ref: REC41/1-024).

### 2.3. Development of the Mobile Application

**Figure 1:**
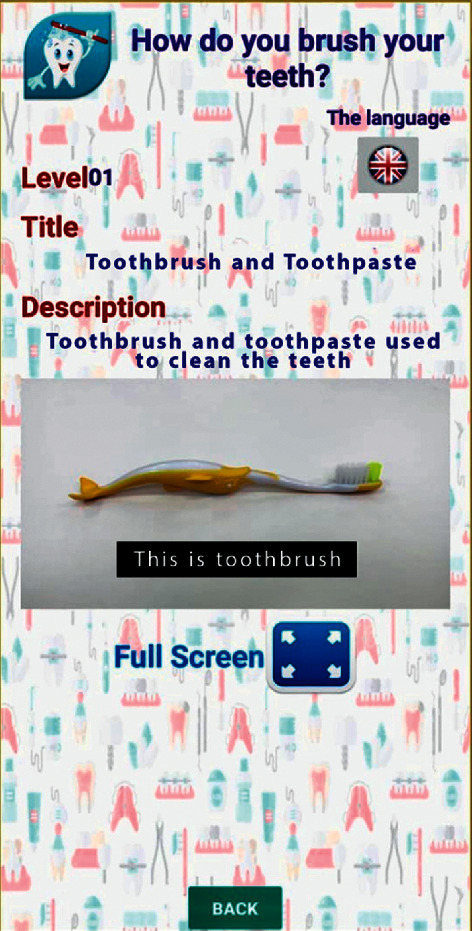
Level 1 showing the basic components of tooth brushing.

**Figure 2:**
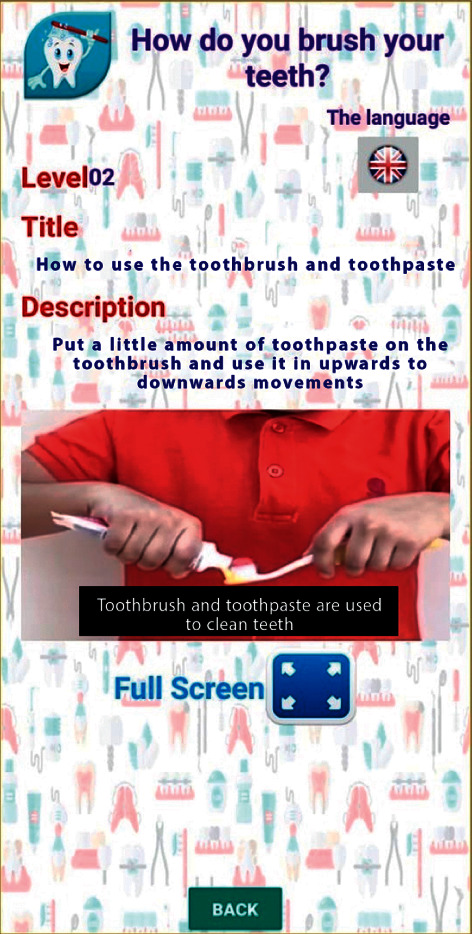
Level 2 showing how to apply toothpaste on the toothbrush.

**Figure 3:**
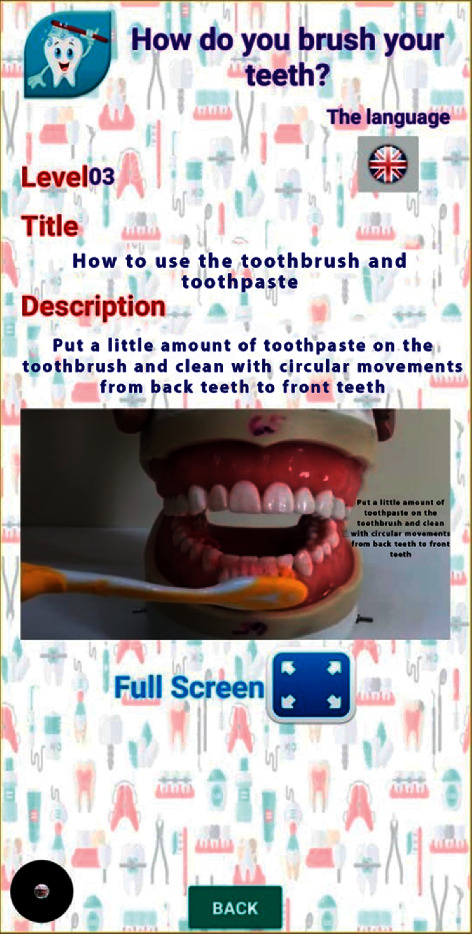
Level 3 showing tooth brushing technique.

**Figure 4:**
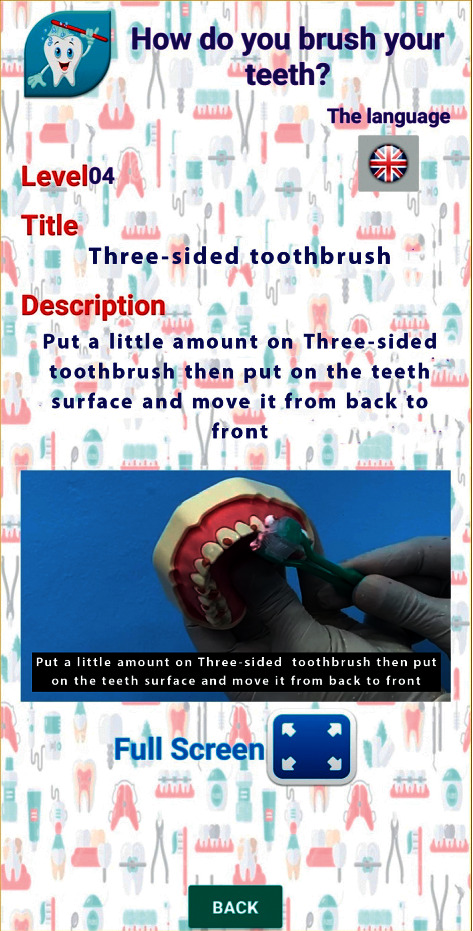
Level 4 showing how to use a special toothbrush for autistic child “Autisticare” on the model.

**Figure 5:**
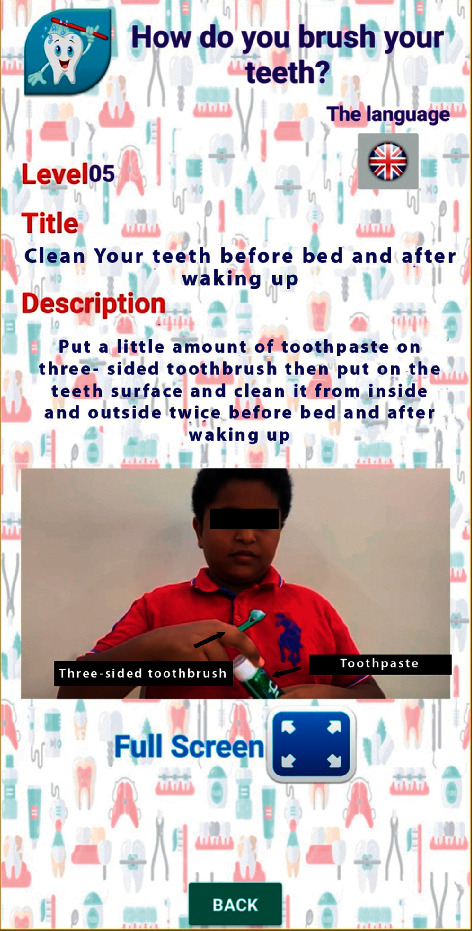
Level 5 showing how to use a three-sided toothbrush in person.

**Figure 6:**
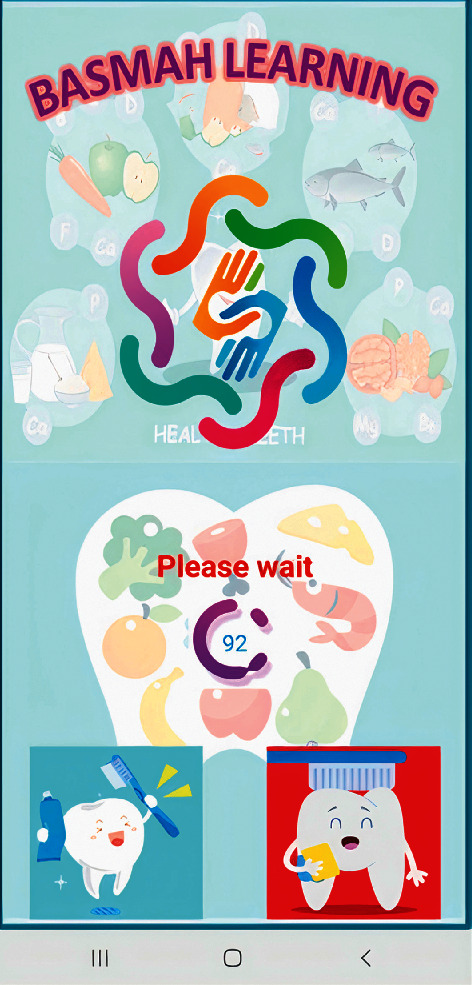
Mobile application “Basmah learning” in the Android play store.

**Figure 7:**
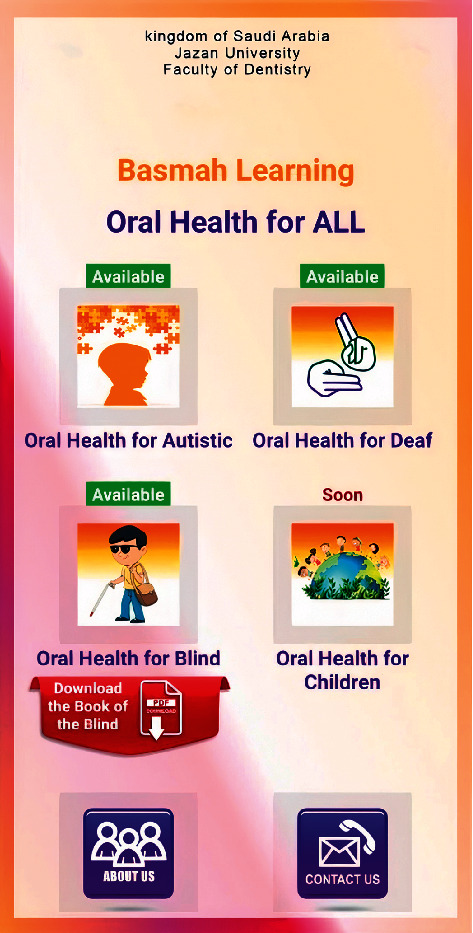
Basmah learning for the deaf, autistic, and blind community.

**Figure 8:**
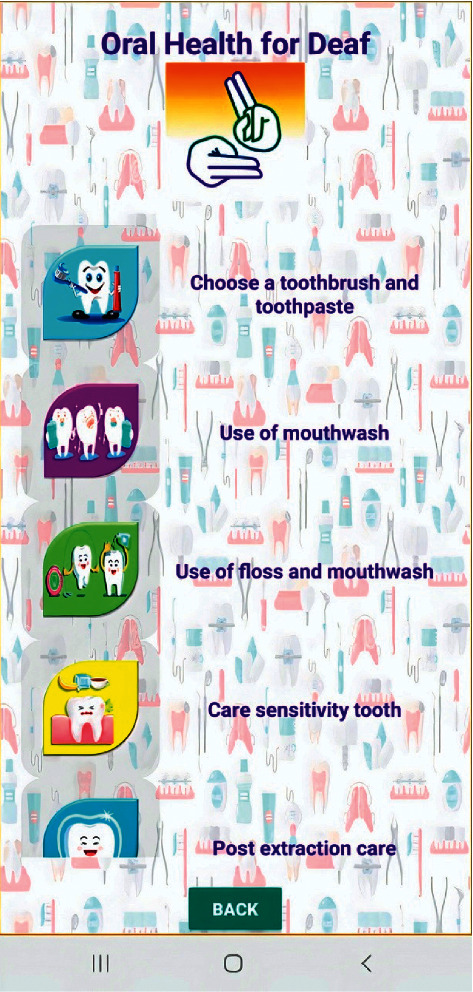
Four categories showing brushing technique, brushing flossing technique, diet, and regular visits.

**Figure 9:**
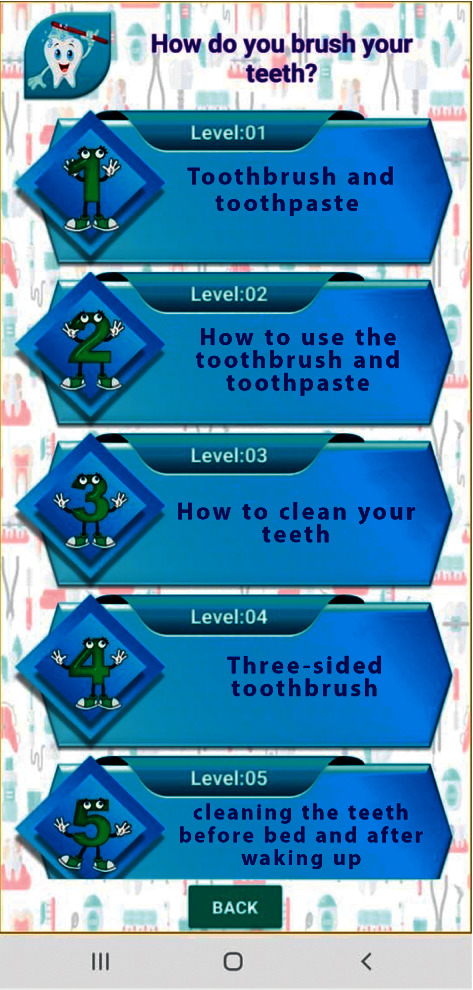
Five levels of tooth brushing technique from level 1 to level 5.

Videos on oral hygiene knowledge and instructions were divided into four main sections: brushing essentials, dental floss, healthy diet, and routine dental visits. Each section was further subdivided into 5 subcategories from level 1 to level 5, wherein the data incorporated was amplified on each level in ascending order (Figures 1–5). Therefore, 15 videos were programmed using Avatar technology depicting various instructions and demonstrations related to oral hygiene knowledge and practices. All videos had voice modulations and running subtitles in the Arabic language for better clarity. While the lower level of each category comprised concise video clips (10–15 seconds) of basic knowledge (Figures 1–3), the larger size videos (30–45 seconds) were of higher levels conveying special brushing (Autisticare) and flossing techniques (Gumchucks) for the autistic children (Figures 4 and 5). Furthermore, a new mobile application, “Basmah Learning,” was custom designed and programmed under the Android play store and IOS Apple store for the disabled community of blind, deaf, and autistic children (Figures 6 and 7). All the custom-designed videos for the autistic children were uploaded under the autistic section of the mobile application (Figures 8 and 9) according to the categories, from level 1 to level 5. All the videos were verified by the autism experts from the autistic school for accuracy prior to the study for ease of understanding by the target audience.

### 2.4. Questionnaire for Assessment of Knowledge

A close-ended questionnaire comprising 14 questions for the autistic children and 21 questions for their parents was designed and translated into Arabic to assess the knowledge, attitude, and practices related to oral health and hygiene. A pilot study was conducted to test and validate the questionnaire (Cronbach's alpha = 0.91). The study participants answered the questionnaire before and after watching the videos in the mobile application. All the questions had multiple choice answers where a correct answer was scored “1” while a wrong answer was scored “0,” hence enabling the calculation of a total knowledge score of each participant.

### 2.5. Study Protocol

All 15 cooperative autistic children received the questionnaire in school while the parents received the questionnaire by Google survey form. The questionnaire-based assessment of their baseline knowledge on oral health hygiene practices was performed before any intervention. The investigator was blinded at all times and was unaware of any allocations or related information of the participants who were involved in the study. An autistic children expert was present with the respondent, who ensured that the participant had fully understood the questions. On completion, the link for the app was sent to the school teachers and the parents of the autistic children. The videos on oral hygiene practices were shown to the children through the application with a gradual update from level 1 to level 5 videos, once every day for four weeks by the teachers and parents. After four weeks, the same set of questionnaire-based knowledge assessment was conducted. The mean knowledge score was then calculated for children and their parents and compared before and after the intervention.

### 2.6. Statistical Analysis

The data was analyzed using IBM SPSS v. 24.0 (IBM Statistics, SPSS, Chicago, USA), and *α* was set at a 5% level of significance. The data were assessed for normality. The frequency and percentage of the responses and the mean knowledge score for the children and parents were calculated. Paired *t*-test was employed to test the difference of the mean before and after knowledge scores. Wilcoxon signed-rank test was used to test the change in the number of participants who answered the questions correctly.

## 3. Results

The present study was conducted among fifteen cooperative autistic children and their parents (*n* = 30) to test the effectiveness of the ABA technique with Avatar technology in improving the knowledge regarding oral hygiene practices. The results of the questionnaires revealed poor knowledge regarding oral hygiene practices among the study participants (Tables 1 and 2). The estimated mean score among the children was 4.73 before the intervention, which significantly increased to 9.0. The estimated mean score for the parents was 9.3 before intervention and 14.6 after a four weeks' period (*P* < 0.0001; Tables 1 and 2).  Table 3 depicts the question wise change in knowledge among the children. All the 15 children demonstrated poor knowledge regarding the number of times to brush the teeth every day and the type of toothbrush bristles to use. Before the intervention, only eight (53%) children provided the correct answers for the number of times they should brush their teeth every day, and only six (40%) children gave the correct answer regarding the type of toothbrush bristles. A significant increase to thirteen (86%) and 9 (60%) after the intervention period was noted.

Similarly, a marked increase was noted in the number of children (four to eight) who demonstrated adequate knowledge regarding the bleeding of gums while using a toothbrush (from 26% to 53%). The participants did not know of any information on the usage of specially designed toothbrushes such as Autisticare or information on specially designed dental floss such as Gumchucks for autistic children. Only two children had an inadequate response (13%) on the use of dental floss; a significant increase in response by nine children (60%) was noted following intervention. Only one child (6%) answered correctly regarding the effect of dietary intake of chocolate, soft drinks, and sugar-containing food on tooth decay, which later increased to six children (46%) after the intervention. Furthermore, only four children (26%) demonstrated limited knowledge regarding routine dental check-ups; this was significantly increased to eleven children (73%) after training.

Table 4 depicts the question wise change in knowledge among the parents. On evaluating parents' responses, the results showed that 65% of the parents helped brush the teeth of their child, which rose to 75% after intervention. Most of the parents noticed the bleeding of the gums while brushing the teeth (85%) and black discolorations on the tooth surfaces (95%), which were reduced to 45% and 80%, respectively, after intervention. The present study also revealed a significant improvement in the knowledge of brushing technique among the parents from initial 5% to 80% and from 0 to 40%. Only 5% of the parents knew about the specially designed three-sided toothbrush for autistic children while 30% of parents knew about the specially designed dental floss “Gumchucks.” However, after the training period, more than 80% of the parents could recall these aids. Around 40% of the parents helped to floss the teeth of their children, which significantly increased to 70%. Regarding dietary habits, only 5% of the parents realized that chocolate, soft drinks, and sugar-containing diets could cause dental decay and pain. Their knowledge significantly improved after the training period. The parents' understanding of the importance of dental visits increased dramatically to 85% from 5%. Approximately 20% of the parents did not attend any special dental awareness programs designed for their autistic kids while 90% of the parents understood the need for specific information for the good oral health of autistic children.

## 4. Discussion

The present study was conducted to apply ABA methods used during behavioral therapy of children with ASD and deliver them through avatars in instructional videos to improve knowledge on oral hygiene practices among cooperative autistic children. The effectiveness of the mobile application in enhancing the knowledge of the children and their parents was assessed using a knowledge questionnaire administered before and four weeks after the training.

Maintaining good oral hygiene in autistic children is difficult due to insufficient interest, hypersensitivity to various stimuli, and lack of necessary manual dexterity. Autistic patients may object to the taste or texture of products such as toothpaste or toothbrush, resulting in poor oral hygiene and a subsequent increase in caries and periodontal conditions [26, 27]. Several authors have reported that patients with autism have poor oral hygiene, and autistic children have more debris and calculus deposits than their counterparts. More debris in these children may be attributed to improper oral hygiene practices, poor knowledge regarding dental and oral care, and an inherent lack of interest in maintaining oral hygiene [27–30]. Hence, the importance of maintaining good oral hygiene in autistic children cannot be overemphasized.

Our research observed that both the children and their parents had a poor understanding of oral hygiene practices. Various researchers have investigated the relationship between the parents' dental knowledge, routine oral hygiene practices, dietary habits, and children's oral health and reported that the better the knowledge and practice of oral hygiene among the parents, the similar is that among their children, and vice versa [14, 31–33]. Moreover, the results of the present study show that the scientifically designed videos using avatars significantly improved the behavior and knowledge of the cooperative autistic children and their parents regarding their oral health and practices. This finding is in accordance with those of AbdAllah et al. who reported a marked improvement in the dental knowledge of caregivers of autistic children in Egypt following a specially designated educational and preventive program for 1 year [34]. Murshid found a specially designed dental book highly effective in enhancing the dental knowledge of parents of children with ASD [18].

The different levels of challenging behavior comprising inattention, aggression, rigidities, and self-stimulation not only add stress to the autistic children's life but also obstruct their learning. Therefore, it is critical to deal with these issues to maximize both effective learning and opportunities [35–37]. A structured sensory stimulation of the autistic child's sensory nervous system is necessary to help them achieve optimum attention to the tasks and perform oral hygiene activities [35]. ABA (Applied Behavior Analysis) technique is a scientifically proven method of treating aberrant behavior with comprehensive, lasting results in autistic individuals [36]. It follows a specific teaching method where the skills are broken down, taught in parts, and built up again. The educational training for the parents in the present study was guided by the study of Metz et al. in 2005, stating that the parent training and support are very critical and a must during ABA technique as the interventions should be initiated at the earliest possible age [37]. Kabot et al. in 2003 emphasized that the intervention should focus on social and communication domains which should be systematic and built upon individualized goals and objectives tailored to the child [38]. Multiple, repeated opportunities are created each day for the child to gain new skills and practice mastered ones. Positive reinforcements are used generously to ensure that the child is motivated [39].

The purpose for designing the mobile application was to fulfill the need for instructions to be scientifically delivered as a structured sensory dose to the autistic children to improve their knowledge about oral health and hygiene practice in Arabic. The application includes an explanation about oral hygiene and demonstrations of tooth brushing technique, flossing, dietary habits, and regular dental visits through videos developed using Avatar technology. Health professionals, caregivers, and teachers can utilize the scientifically designed mobile application in order to facilitate communication with cooperative autistic children and their parents.

Limitations of the current study include the following: limited access to children with autistic disorder, the exclusion of uncooperative subjects, and a small sample size due to difficulty in recruitment of subjects during the COVID-19 pandemic. Additionally, the demographic status of the parents such as their educational level was not considered. Further longitudinal studies are required in order to assess behavioral changes among autistic children following the long-term use of the mobile application. It is recommended that the application is translated to other languages in order to help similar target groups in other countries.

## 5. Conclusions

It can be concluded from the present study that structured sensory doses of oral hygiene instructions delivered in the form of videos that apply behavior analysis methods can improve knowledge regarding oral hygiene practices among cooperative autistic children.

## Figures and Tables

**Table 1:** Comparison of mean knowledge score before and after training in autistic children (before and after receiving the instructions).

Time	*N*	Mean	±SD	Mean difference	“*t*” value	*P* value
Before training	15	4.733	1.751	4.267 ± 2.789	5.924	<0.001^*∗∗*^
After training	15	9.000	2.138			

^#^Student's *t*-test unpaired ^*∗*^*P* < 0.05, significant; ^*∗∗*^*P* < 0.001, highly significant.

**Table 2:** Comparison of mean knowledge score before and after training in parents of the autistic children (before and after receiving the instructions).

Time	*N*	Mean	±SD	Mean difference	“*t*” value	*P* value
Before training	20	9.300	2.716	5.300 ± 3.541	6.694	<0.001^*∗∗*^
After training	20	14.600	2.393			

^#^Student's *t*-test unpaired; ^*∗*^*P* < 0.05, significant; ^*∗∗*^*P* < 0.001, highly significant.

**Table 3:** Questionwise response (correct) in children (*N* = 15).

S. no	Question	Before		After		*Z* value#	*P* value#
		*N*	%	*N*	%		
1	How many times do you brush your teeth daily?	8	53.3	13	86.7	1.890	0.059
2	How much time you spend on brushing?	8	53.3	6	40	0.707	0.480
3	Do you know about special toothbrush which is “3-sided toothbrush” (Autisticare) especially designed for autistic children?	0	0	12	80	3.464	0.001^*∗*^
4	Do you use dental floss?	2	13.3	9	60.0	2.333	0.020^*∗*^
5	Do you know about special dental floss by name “Gumchucks” especially designed for autistic children?	0	0	12	80.0	3.464	0.001^*∗*^
6	Have you ever tried to notice any black discoloration/spots on your tooth?	10	66.7	9	60.0	0.378	0.705
7	Visit to the dentist will improve the oral health?	9	60.0	12	80.0	1.342	0.180
8	Do chocolate, soft drinks, and sugar-containing food cause tooth decay and tooth pain?	1	6.7	7	46.7	2.121	0.034^*∗*^
9	Did you ever try to notice bleeding from gum while brushing your teeth?	4	26.7	8	53.3	1.414	0.157
10	What type of toothbrush you should use for brushing your teeth?	6	40.0	9	60.0	1.000	0.317
11	How many times you should visit the dentist for regular check-up?	4	26.7	11	73.3	2.646	0.008^*∗*^
12	What is the effect of fibres containing vegetables and fruits on general health of the teeth?	6	40.0	6	40.0	0.000	1.000
13	Do you think eating sweet food is good for teeth and oral health?	10	66.7	11	73.3	0.447	0.655
14	Which vitamin is good for the gums of the teeth?	3	20.0	10	66.7	2.333	0.020^*∗*^

^#^Wilcoxon signed rank test; ^*∗*^*P* < 0.05, significant; ^*∗∗*^*P* < 0.001, highly significant.

**Table 4:** Question wise response (correct) in parents (*N* = 20).

S. no	Question	Before		After		*Z* value#	*P* value#
		*N*	%	*N*	%		
1	Do your clean your child teeth using toothbrush and toothpaste?	13	65	15	75	0.632	0.527
2	Have you noticed bleeding from gum while your child is brushing his teeth?	17	85	9	45	2.309	0.021^*∗*^
3	How many times your child should brush the teeth daily?	1	5	16	80	3.873	<0.001^*∗∗*^
4	How long your child should spend on brushing the teeth?	0	0	8	40	2.828	0.005^*∗*^
5	Do you know about special toothbrush which is “3-sided toothbrush” (Autisticare) especially designed for autistic children?	1	5	16	80	3.873	<0.001^*∗∗*^
6	Have you ever tried to notice the black discoloration/spot in your child tooth?	19	95	16	80	1.732	0.083
7	Are you satisfied about your child's dental health?	10	50	13	65	1.000	0.317
8	Do you floss your child's tooth with the dental floss?	8	40	14	70	1.897	0.058
9	Do you know about special dental floss by name “Gumchucks” especially designed for autistic children?	6	30	17	85	3.051	0.002^*∗*^
10	What is the effect of fibres containing vegetables and fruits on general health of teeth?	8	40	15	75	2.111	0.035^*∗*^
11	Do you think eating sweet food is good for teeth and oral health?	14	70	9	45	1.667	0.096
12	Do chocolate and sugar-containing food cause tooth decay and tooth pain?	1	5	13	65	3.464	0.001^*∗*^
13	Do you think drinking soft drink can cause tooth decay, tooth pain, or gum disease?	11	55	10	50	0.333	0.739
14	Did you visit the dentist before for your child's dental check-up?	1	5	17	85	4.000	<0.001^*∗∗*^
15	How many times you should visit the dentist for regular check-up of your child?	13	65	16	80	1.000	0.317
16	Is special information necessary about dental care for autistic child?	10	50	18	90	2.309	0.021^*∗*^
17	Have you seen any awareness programs for autistic child dental health?	4	20	14	70	2.887	0.004^*∗*^
18	Does your child chew the pencil and injure the gums?	10	50	17	85	2.111	0.035^*∗*^
19	Do you know about special “pencil toppers” made for autistic child to prevent the accidental gum injury due to pencil chewing?	19	95	19	95	0.000	1.000
20	Does your child have the habit to bite the nail and fingers and injure the gums?	16	80	8	40	2.530	0.011^*∗*^
21	Do you know about special “chew necklace” device made for autistic child to prevent the gum injury by nail/finger biting?	4	20	12	60	2.530	0.011^*∗*^

^#^Wilcoxon signed rank test; ^*∗*^*P* < 0.05, significant; ^*∗∗*^*P* < 0.001; highly significant.

## Data Availability

The SPSS data file used to support the findings of this study are available from the corresponding author upon request.
